# Odontalgia

**Published:** 1892-05

**Authors:** Charles P. Briggs


### ODONTALGIA.[1]

BY CHARLES P. BRIGGS, M.D., D.M.D.

THIS paper is intended to be an enumeration of the causes of pain referred to the teeth, with especial regard to the differential points of diagnosis. Little can be said on this subject which has not been said before; it is hoped that the profit may be found in the relation of experiences.

It is not of infrequent occurrence for a patient to come complaining of toothache; in the majority of cases, from one's records, or from unmistakable symptoms, it is made evident, in a very short time, just where the trouble is. Occasionally, however, the diagnosis becomes extremely difficult, and one has to consider all the causes of pain, and reach his diagnosis through elimination.

### SURFACE SENSITIVENESS.

One writer says, "In very rare instances an apparently sound and healthy tooth will become the seat of severe and continuous pain, which, for the time, resists amelioration from the application of any of the remedies usually employed. Generally, however, such a tooth, if the intensity of the pain does not necessitate its removal, will be found within a limited period to be responsive to local irritants, such as sweet, sour, hot, or cold articles, when brought into contact with some circumscribed part upon the crown, the response manifesting itself as acute but temporary pain. The cause of this morbid condition is but imperfectly understood, and its pathologi-

---

[1] Read at a meeting of the Harvard Odontological Society, November 19, 1891.

cal significance is only fully appreciated when the sensitive surface yields to some destructive agent, resulting in molecular disintegration or caries." Such a spot upon the crown of a tooth must, as this author says, be very rare; but it is by no means uncommon for spots upon the roots of teeth, when there has been more or less recession of the gums, to become thus sensitive, and responsive to a painful degree to the sort of irritation just mentioned. Especially is this liable to occur when the relation of adjoining teeth is such that food is allowed to crowd up between the teeth, where, by its macerating effect and by the action of the acids formed in its fermentation, it brings about this hypersensitiveness of the surface. That this is the cause is proven by the fact that the sensitiveness disappears when, owing to the presence of approximal cavities, it is possible so to contour the teeth that food shall not be wedged up between them. Pain due to this cause is pretty definitely located by the patient, and responds to the point of the examining instrument. That there is no depression or roughness of the surface shows that the condition is not the one next to be mentioned.

### CARIES.

Caries, though superficial, may be accompanied by pain wholly out of proportion to the loss of tooth-substance. If caries is the cause of pain, in a given case, it is made evident by the unusual sensitiveness of the spot to the blade of the excavator, as well as by the history of pain in that region after eating sweets and acids.

That the pain from this cause may be distressing and continuous, I can myself bear witness. While eating sweets, I began to feel a pain in an upper wisdom-tooth, which had never given me any trouble. The pain, soon becoming excruciating, lasted an hour. There was no question in my mind but that the pulp was exposed. Examination, however, showed that the caries—the cause of the pain—was very superficial indeed.

### HYPERÆMIA AND INFLAMMATION OF THE PULP.

When one considers how narrow is the line between hyperæmia and inflammation (in the one case a congestion of the capillaries of the pulp; in the other, the same condition with the migration of the corpuscle and the exudation of lymph added), it is found that the diagnosis between the two conditions (hyperæmia and inflammation) is difficult or impossible. That it is necessary to distinguish between the two conditions is owing to the fact that, if the case be one of simple hyperæmia, there may be a good chance of saving the pulp by improving the conditions of the tooth.

Hyperæmia may occur in a tooth apparently sound; more frequently it is found in one in which there is a cavity of considerable depth, or a filling of large size, thus subjecting the pulp to changes of heat and cold. The pain of these two conditions is, pretty generally, definitely located by the patient.

In hyperæmia, the pain is paroxysmal, of a lancinating, excruciating character, and is usually induced by thermal changes. In the intervals between the paroxysms there may be complete cessation of pain.

The same symptoms hold for inflammation of the pulp, except that the pain is not as severe, as a rule, and is more continuous. The pulp does not respond quite as readily to heat and cold.

A convenient and effective way of testing a tooth, to see whether it is responsive to thermal changes, may be mentioned here. A piece of ice, to give a satisfactory result, needs to be small enough to cover but one tooth. When thus small, it is hard to handle, melting before the cold has had time to penetrate the walls of the thicker teeth. On the other hand, heat from an ordinary instrument, as, for example, the ball burnisher, is dissipated too quickly to afford a conclusive test. When an Evans root-dryer is kept in the bunsen-flame till the bulb is red-hot, enough heat is liberated at the tip of the silver point to scorch the fingers as it is quickly drawn over them. Moreover, the heat is kept up to this high point for some little time. If there is any pulp alive in a tooth in question, the tip is pretty sure to find it out. This can be applied, too, at the thinnest part—the neck—without danger of burning the gum.

### PERICEMENTITIS.

The prominent symptoms of all inflammation of the pericementum is tenderness on percussion and on movement of the tooth. Pain varies according as the cause of the trouble takes its origin from the margin of the gum, or from the apex of the root. A wedge pressing upon the gum, or the presence of fermenting food in a space between two teeth not easily reached, or a deposit of tartar, may set up a pericementitis. There is some lameness of the teeth involved. The pain, in most instances, amounts to nothing more than a "grumbling." When the trouble is at the apex of the root, however, from extension or inflammation of the pulp, or from the ingress of septic material through the empty canal, the pain, at first dull, soon becomes severe, throbbing, and unendurable. There is great tenderness on percussion, together with a lifting of the tooth in its socket. Heat does not aggravate, unless after long application, and then to a moderate degree. Cold, on the other hand, brings relief.

An interesting point to be mentioned here is, that in teeth with more than one root, a pericementitis may be set up on one root at its apex, while another root is still alive. Forgetting this, it would be very confusing to drill into a tooth which gave all the signs of pericementitis, and find the tooth sensitive.

### NODULAR DENTINE OR PULP-STONES.

This condition is by no means uncommon. The diagnosis, as there are no distinctive symptoms, is not easy, and must be reached by exclusion. The pain is neuralgic in character, and, at first, is not apt to be attributed to the teeth. If attention is called to them, there will probably be found a slight tenderness on percussion in the tooth which is the seat of the trouble. The neuralgic pains are paroxysmal, there being pretty complete relief from pain during the intervals.

### HYPERCEMENTOSIS.

What has been said concerning nodular dentine applies almost word for word to hypercementosis. Pain, if associated with the teeth, is neuralgic in character. The affection is much more rare, however, than nodular dentine, or, at all events, its presence is discovered less frequently by the practitioner.

### UNERUPTED OR IMPACTED TEETH.

Such teeth may be the cause of mysterious toothache. In rare cases, inflammation of the pulp and pericementum may be set up in unerupted teeth. Impacted inflamed wisdom teeth, from their position, pressed down upon the nerve-trunk, are very liable to give rise to severe toothache, with neuralgia, trismus, and anchylosis.

### INFLAMMATION OF THE ANTRUM.

Inflammation of the mucous membrane of the antrum when connected with the teeth, as regards the etiology, or as far as the symptoms are concerned, is due, in most instances, to an extension of an inflammation of the pericementum of some of the teeth which not infrequently extends into that cavity. Occasionally, however, as Garretson mentions in his "System of Oral Surgery," a purulent inflammation of the membrane which, in its origin, is independent of the teeth may be characterized by neuralgic pains, with a localization in the bicuspid or molar teeth.

A case of antral abscess I have in mind was as follows: The patient complained of agonizing pain in the left upper second bicuspid, in which there was a small distal cavity filled with gutta-percha. The filling was removed. Being found in good condition, the cavity was refilled. It was concluded that the case was one of simple hyperæmia, and, after the application of iodine to the gum, the patient was dismissed, with the hope that the pain would become quiet.

The pain continuing, relief being demanded, the pulp was devitalized, removed, and an antiseptic dressing left in the root. No relief coming from this procedure, a drill was put up into the antrum, giving vent to the pus confined there.

At the outset there are no diagnostic points of this affection, unless earache and ringing in the ears (which are pretty constant symptoms) be mentioned. Later, when pus has formed, there is a feeling as of the running of water when the head is moved from side to side, together with the escape of pus into the nostril of the affected side.

### REFLEX TOOTHACHE.

In a case of toothache for which it is difficult to find a cause, the possibility of its being a reflex expression of trouble in some other part of the body, or in some other tooth, must not be forgotten.

As an illustration of the very common reflex toothache of the superior second bicuspid when the inferior wisdom-tooth is at fault, and as an example of the fact that one should never rely upon "subjective" symptoms, nor be deceived by appearances in making a diagnosis, the following case is given:

A patient came to me, saying that her teeth had recently been examined by her dentist, who had pronounced their condition good. She had been suffering from pain in the right superior second bicuspid, the pulp of which she thought had been capped. As the filling—a soft gutta-percha—was leaky, it was removed. The cavity was very deep, and, in most cases, there would have been an exposure of the pulp, but there had evidently been a recession of the pulp, with the deposit of secondary dentine. As the floor of the cavity was hard, and the smallest probe could find no opening into the pulp-chamber, it was determined to fill again, putting an antiseptic dressing at the bottom, gutta-percha and cement on that. All went well for a month, when the patient returned, saying that the pain was worse than before. An exposure of the pulp was made. The pulp was paralyzed with cocaine, removed, and the roots filled with gutta-percha. The patient was dismissed with the assurance that her pain was at an end. She returned in a few days, saying that she had been in agony. Here was a disappointment for me, a believer in root-filling, for in this case all the conditions had been favorable. Percussion, however, showed that the bicuspid was absolutely without tenderness.

Examination of the lower wisdom-tooth revealed a cavity. This was excavated and dressed, and there was an end of the pain.

Pain may be reflected from more distant starting-points. Slight operations in the nasal passages, such as the removal of adenoid growths and the cauterization of hypertrophied mucous membrane, covering the turbinated bones, may be followed by toothache. Diseases of the eye and ear, of the stomach, of the uterus, and the condition of pregnancy may be accompanied by pain in the teeth.

A case which may be mentioned here as illustrating how the sensation of pain may be reflected from its proper source was seen by me a few days ago.

The patient, a woman, began to be troubled five years ago by neuralgia in different parts of the distribution of the trifacial. The teeth, however, had never been the seat of pain. During all that time she had been under treatment, free from pain, except under the influence of analgesics. The pain having become localized in her ear, she was sent to an aurist. He, finding no trouble, sent her to a neurologist, who, after a careful examination, told her that the only thing to do before availing herself of the last resort (excision of a portion of the nerve) was to consult a dentist. He brought her to Dr. Hamilton, who, finding an exposure of the pulp in a lower third molar, ordered the tooth extracted. In his absence, I saw the patient, when, two days later, she came to report. For the first time in five years, she said, she had been entirely free from pain. She left, filled not with a spirit of thankfulness that her sufferings were over, but with a desire for vengeance upon the men who had kept her under treatment so long, when the trouble was all due to a wisdom-tooth.

In cerebral diseases and hysteria, a patient may complain of toothache when the teeth are in no way at fault.

### SYSTEMIC DISEASES.

Finally, syphilis, malaria, rheumatism, and gout may be mentioned as among the rare causes of toothache.

